# Pseudo-Random Number Generator Based on Logistic Chaotic System

**DOI:** 10.3390/e21100960

**Published:** 2019-09-30

**Authors:** Luyao Wang, Hai Cheng

**Affiliations:** Electronic Engineering College, Heilongjiang University, Harbin 150080, China; 2181244@s.hlju.edu.cn

**Keywords:** logistic chaotic system, PRNG, Pseudo-random number sequence

## Abstract

In recent years, a chaotic system is considered as an important pseudo-random source to pseudo-random number generators (PRNGs). This paper proposes a PRNG based on a modified logistic chaotic system. This chaotic system with fixed system parameters is convergent and its chaotic behavior is analyzed and proved. In order to improve the complexity and randomness of modified PRNGs, the chaotic system parameter denoted by floating point numbers generated by the chaotic system is confused and rearranged to increase its key space and reduce the possibility of an exhaustive attack. It is hard to speculate on the pseudo-random number by chaotic behavior because there is no statistical characteristics and infer the pseudo-random number generated by chaotic behavior. The system parameters of the next chaotic system are related to the chaotic values generated by the previous ones, which makes the PRNG generate enough results. By confusing and rearranging the output sequence, the system parameters of the previous time cannot be gotten from the next time which ensures the security. The analysis shows that the pseudo-random sequence generated by this method has perfect randomness, cryptographic properties and can pass the statistical tests.

## 1. Introduction

With the rapid development of communication technology and the wide use of the Internet and mobile networks, people pay more and more attention to information security. The primary reference source for protecting data is cryptology [1]. Because of its high performance, the chaotic system has attracted a lot of attention from researchers [2].

There is an interesting relationship between chaos and cryptography. According to this relationship, many characteristics of chaotic systems such as the sensitivity of initial value/system parameters, ergodicity, deterministic dynamics, and structural complexity are considered to be the diffusion and confusion of keys [3,4,5,6]. Compared with traditional encryption systems, they have certain randomness [7,8]. As a result of this close relationship, several chaos-based cryptosystems have been put forward since 1990 [9].

On the one hand, the extreme sensitivity of chaotic systems to initial conditions makes chaotic systems very attractive for cryptographic applications, especially for pseudo-random number generators (PRNGs) [7,9,10,11,12,13,14,15,16,17,18,19,20,21]. Several chaotic systems have been successfully used [8] and play an important role in many applications such as numerical simulations, the game industry, communication and stochastic computation [22]. On the other hand, PRNG is an important module in the development of cryptosystems to be robust against different types of security attacks. Some PRNGs have been implemented in personal computers and in embedded systems [4,23]. A PRNG is defined as an algorithm that can produce random number sequences whose main advantage is the rapidity and repeatability of a generating process, which is one of the most important components in stream cryptography [20]. In practical applications, the generation is not trivial, and the randomness of generated sequences is the key to the selection of applications [7].

Logistic map is one of the most commonly used chaotic map in chaotic cryptography for it is one of the simplest and most studied nonlinear systems [24] and has been widely used in block ciphers, stream ciphers and hash functions. In 1947, Ulam and von Neumann proposed a PRNG based on the logistics map [25]. Oishi and Inoue designed a PRNG by using chaos first order nonlinear difference equation in 1982 as well as construct a uniform random number generator with arbitrary Kolmogorov entropy [17]. In 1999, Gonzalez and Pino put forward a truly unpredictable random function to help generate real random numbers by extending logistic map [18]. Wang et al. realized the binary sequence of chaotic orbit with limited computational accuracy which was based on z-logistic mapping in 2006 [19]. In 2009, Patidar [9] and Patidar et al. [7] proposed a novel pseudo-random bit generator based on two chaotic logistic maps and two chaotic standard maps, respectively. The chaotic maps are running side-by-side and starting from random independent initial conditions. Their schemes were studied for the NIST (National Institute of Science and Technology) and DIEHARD tests suites, which are considered the most stringent statistical tests suites for randomness [26]. In 2013, François M et al. presented a PRNG algorithm based on mixing three chaotic maps produced from an input initial vector [13] which uses the standard chaos function and linear congruence to calculate and index the arranged position and passes the test of the NIST test suite. In the raised schemes, many researchers have proposed similar feedback approaches [9,27,28] such as Patidar achieved the continuous operation of PRNGs by using the value generated by the previous chaotic system as the initial value of the next iteration of chaotic systems [9]. In 2016, Xu D et al. proposed separate chaotic mapping from linear feedback shift register (LFSR), where the results of chaotic iteration are quantified after the binary bit sequence is reached, xor is performed with the m sequence outputted by the LFSR to realize the disturbance, and finally the disturbance result is returned to the input of the chaotic iterative system for the next round of iterative operations [14]. In the PRNG based on the piecewise logistic chaotic system proposed by Wang et al., the parameter m is used to limit the system parameters of a chaotic system and adjust its next iteration to remedy the randomness problem [20].

At the present stage, the research of PRNG based on chaos focuses on the complexity of random bit extraction which attaches importance to reduce the possibility of extracting chaotic information by improving the complexity of the algorithm. Because all chaotic systems are deterministic systems, chaotic behavior can be identified by some methods in chaos theory [22] which means it is not safe to design PRNG with fixed system parameters. The idea of feedback is used to realize the continuous output of pseudo-random numbers, but the correlation between the previous and next is the key to security. An algorithm is proposed on the basis of the logistic chaotic system, which can realize the iteration of the pre-and-post chaotic system and the independence of the pre-and-post iteration. The change of parameters of the chaotic system in the algorithm has no statistical regularity, then realizes the generation of the pseudo-random sequence would be safe enough from the point of view of the chaotic system. After the analysis, it was found that it had good randomness and no obvious statistical information.

## 2. Pseudo-Random Sequence Generator Algorithm

The process of the PRNG’s generation in this paper can be divided into initial state and normal state. Seed parameters need to be selected manually in the initial state [29], while seed parameters need not be selected manually in the normal state. The selection of normal seed parameters is related to the generation of antecedent pseudo-random sequences.

### 2.1. Generation of Initial Pseudo-Random Sequences

Select the initial seed parameters $\left(\mu_{1},x_{0_{1}},I_{1}\right)$, where $\mu_{1}$ is the system parameter, $x_{0_{1}}$ is the initial value and $I_{1}$ is the number of iterations. [3.5699,4] is the value range of $\mu_{1}$, [0.0,0.5] is the value range of $x_{0_{1}}$, more than 1000 is required to improve the randomness of the sequence $I_{1}$. The value of the initial seed parameter is arbitrary.

According to logistic chaos equation, we can get 16 floating point numbers $y_{1}$ by iterating for $I_{1}$ times.

Discarding the integer part of $y_{1}$ could get a 15-decimal number, respectively arbitrary select 3 digits to form a 3-decimal number, 4 digits to form a 4-decimal number, 5 digits to form a 5-decimal number, all the way up to 15 digits to make up a 15-decimal number. A whole numerical sequence is obtained by taking each decimal number module 256 and converting it into a binary sequence, which is a pseudo-random sequence generated at the initial state. Also, 104 numbers of pseudo-random sequences can be generated by one chaotic iteration.

### 2.2. Generation of Normal Pseudo-Random Sequences (Taking the Nth Times as an Example)

Retain the fractional part of the floating point number $y_{N-1}$ generated at the (N−1)th, thus a 15 decimal number can be got and then rearrange it for once time. If the different number of positions after rearranging between the previous and next time is less than 12, it will be rearranged again until the condition is met. The 1st, 5th, 9th and 13th numbers are taken to form the decimal part with the integer part 0 as the initial value $x_{0_{N}}$ of the chaotic system.

**Figure 1:**
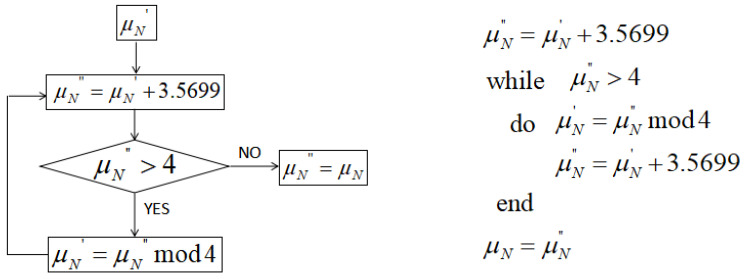
Parameter generation of normal chaotic system.

The 2nd, 6th, 10th, and 14th digits are taken as the preparatory system parameter $\mu_{N}^{\prime }$ of the chaotic system. The flow chart of the generation of $\mu_{N}$ is shown in Figure 1. The algorithm description is also given together.

**Figure 2:**
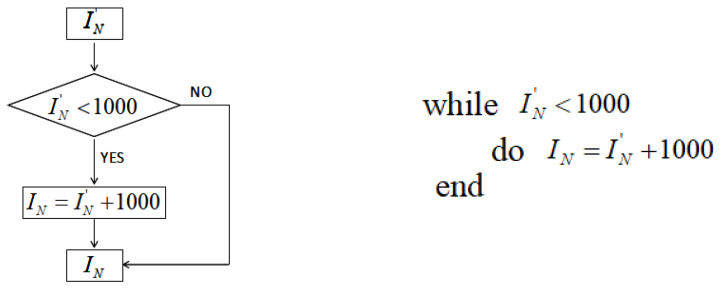
Iteration times of the normal chaotic system.

The 3rd, 7th, 11th, and 15th digits are taken to form the 4-bit integeral $I_{N}^{\prime }$. To be within a better range, the flow chart of the iteration number $I_{N}$’s generation is shown in Figure 2. The algorithm description is also given.

## 3. Performance Analysis

In this chapter, different perspectives will be discussed concerning the proposed scheme, including the logistic chaotic system, algorithm running process, safety analysis and differential analysis [2].

### 3.1. Logistic Chaotic System

Logistic chaotic system is a classical chaotic system, thus it has been widely used in data security and secure communication due to its complex dynamic behavior [3,24]. The mathematical expression of logistic equation is:

(1) $$X_{n+1}=\mu X_{n}\left(1-X_{n}\right),n=0,1,2,3\cdots$$

μ is the system parameter of the logistic equation, and the initial value of the system is set to $x_{0}\left(0<x_{0}<1\right)$. When 3.5699<μ<4, the system goes into chaos [9,20].

**Figure 3:**
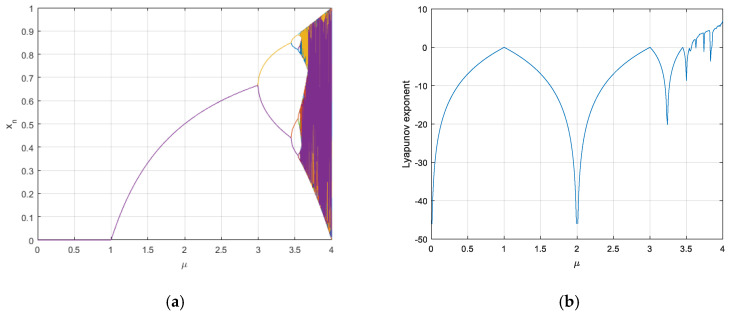
(**a**) Logistic bifurcation diagram; (**b**) Lyapunov exponent of Logistic mapping.

As shown in Figure 3a, with the increase of μ, the system gradually enters into a chaotic state [20]. Figure 3b shows the Lyapunov exponent of logistic mapping when 3.5699<μ<4, the Lyapunov exponent is positive, and it can be determined that the system is in a chaotic state at this time [9]. As an inspiration, it is necessary to ensure that the parameter interval of the designed key flow generator is in a chaotic state, adopted system would have typical basic characteristics of chaos under this condition [24]. The selection of parameters in the algorithm can guarantee the μ during each cycle operation located in the interval [3.5699,4], and the chaotic state of the system could be emerged without any accidents [22].

Logistic chaotic system is extremely sensitive to initial values. No matter how close the two points are, orbits of logistic chaotic system may be separated under the effect of mapping on account of the extreme sensitive to initial value [13]. Table 1 shows that a very slight change of the initial value can lead to a great difference and the sensitivity of the logistic mapping to the initial value is very significant [20].

**Figure 4:**
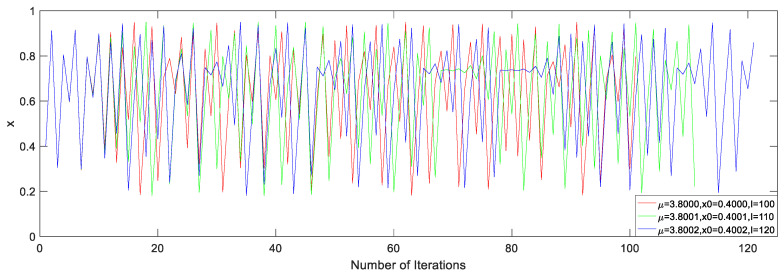
Dynamic behavior under different seed parameters.

Under the different system parameters μ, initial values $x_{0}$, and iteration numbers I, the dynamic behaviors greatly differ from each other, which can be seen in Figure 4.

The 16 floating point numbers generated with different seed parameters are shown in Table 2 [30,31]. The first three rows of data in Table 2 correspond to the results under seed parameters shown in Figure 4.

The above analysis of logistic chaotic system shows its sensitive dependence and unpredictability to initial value; the minor change in the parameters could result in a great change [32,33,34]. Although these values are limited between bounds, they are pseudo-random and do not converge after any value of iterations [24].

### 3.2. Sequence Analysis

The 15 number sequence would be generated by chaotic system in the proposed algorithm, then selects 3 digits to form a 3-decimal number randomly, 4 digits form a 4-decimal number and 5 digits form a 5-decimal number, all the way up to 15 digits making up a 15-decimal number. There are 13 integers in total:

$$A_{15}^{3}+A_{15}^{4}+\cdots +A_{15}^{15}\approx 3.55\times 10^{12}$$

$A_{n}^{m}$ represents an arrangement of extracting m elements from n different elements. A full numerical sequence is obtained by modulating each decimal number to 256. Obviously, all values in this sequence are located in [0,255]. Several aspects are analyzed when seed parameters $\left(\mu ,x_{0},I\right)$ equal to (3.8000,0.5000,1000).

#### 3.2.1. Rearrangement Analysis

Seed parameters $\left(\mu ,x_{0},I\right)$ of the next chaotic system are extracted and formed after the rearrangement of the 15 real numerical sequence generated by the chaotic system in the proposed algorithm. The function of rearranging is to increase the sequence space and reduce the correlation between the previous chaotic behavior and the next. If the similarity between the rearranged sequence and the original sequence is large, the purpose is not achieved. This situation is considered in two ways:

- In the 15 digital sequence generated by chaotic sequence, a single real number iterates many times and more than one number iterates many times.
- The different numbers between the rearranged sequence and the original sequence are less than 12.

**Figure 5:**
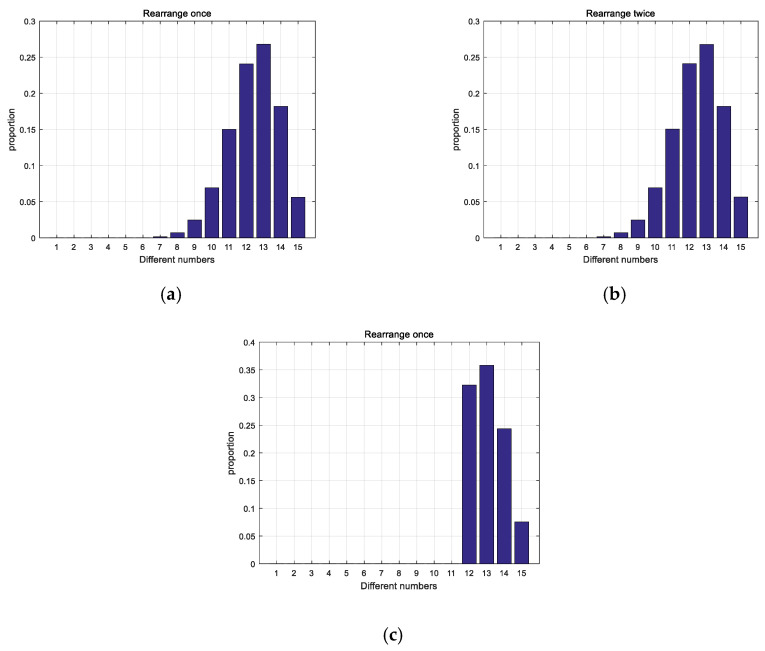
Comparison of distribution: (**a**) Distribution after rearranging once; (**b**) Distribution after rearranging twice; (**c**) Distribution after improved.

As shown in Figure 5a,b, comparing the rearranged sequence and the original sequence for $10^{7}$ times, the increase of the number of rearrangements cannot improve the difference degree no matter the times of rearranging. The distribution after limiting the degree of difference is shown in Figure 5c. Obviously it can be seen from the figure that the difference of the sequences has been improved, thus the correlation has been reduced.

#### 3.2.2. Extraction Analysis

The information entropy [2] under different extraction methods for repeated extraction several times is shown in Table 3. The extraction method in Table 3 represents the number in the 15-digit numerical sequence.

Table 3 shows the proposed method has little effect on the randomness. In this paper, the last three extraction methods in Table 3 are selected to extract the initial value, system initial value and iteration times of the next chaotic system.

#### 3.2.3. Modular Operation Analysis

(1) Distribution Analysis

The algorithm is run on the basis of the seed parameters (3.8000,0.5000,1000), and the generated seed parameters are shown in Table 4.

**Figure 6:**
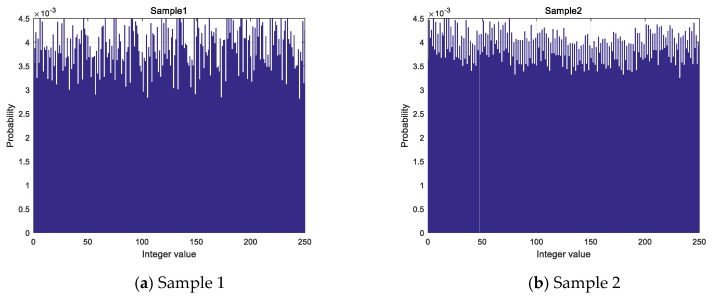

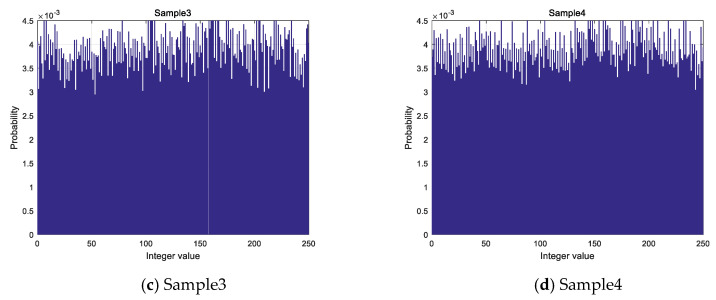
Histogram of integer value sequence: (**a**) Histogram of integer value sequence of sample 1; (**b**) Histogram of integer value sequence of sample 2; (**c**) Histogram of integer value sequence of sample 3; (**d**) Histogram of integer value sequence of sample 4.

According to the algorithm, a whole numerical sequence is obtained by taking each decimal number modulo 256. By analyzing the sequence generated under the samples, the distribution is shown in Figure 6. The transverse coordinates in the graph represent the values of the obtained integer value sequence and the longitudinal coordinates represent the probability of the occurrence of the values.

The standard deviation of each sample is shown in Table 5 which represents the degree of discretization of the data, and the standard deviation represents a large difference between most of the values and the average value. The distribution of 256 values in integer value sequence is not absolute average combined with the results of the distribution histogram and standard deviation, but its distribution is different from the Gaussian distribution, which is particularly prominent in some values, so it is considered that the distribution of values in integer value sequence is more uniform.

(2) Entropy Analysis

High values of entropy mean a robust PRNG, whereas low values of entropy mean a weak PRNG with a certain degree of predictability [26]. Information entropy is a quantitative metric measuring the disorder or randomness of integer value sequence. The more chaotic a sequence, the higher the information entropy [1,2]. When the distribution of sequence values is an equal probability distribution, that is, when the probability of each value between [0,255] is 1/256, it has the maximum entropy of lg256=8 bit [5]. In Table 6, sample points were extracted for information entropy calculation according to the experimental samples in Table 4.

It can be seen from Table 6 that the information entropy of randomly extracted sample points is close to the expected value of 8. It is demonstrated that the integer value sequence extracted by this algorithm has strong uncertainty, good randomness, and no clear statistical information.

#### 3.2.4. Analysis of Pseudo-Random Sequences

In order to ensure the security of the cryptosystem, a good cryptosystem must be sensitive to the key [2,5,13,35,36,37]. The chaotic sequence is very sensitive to the seed parameter $\left(\mu ,x_{0},I\right)$, and its dynamic behavior is shown in Figure 4. The data in Table 2 are modified to make minor changes to one or more parameters of the chaotic system to generate new binary sequences. Let p as a different number between the previous and the next sequence, q as the length of the sequence, the ratio r=p/q [15] is calculated and shown in Table 7. It can be concluded from the data in the table that the generated sequence is sensitive to seed parameters.

### 3.3. Safety Analysis

#### 3.3.1. The Key Space

A necessary condition for an encryption scheme to be secure is that the key space is large enough so as to frustrate brute-force attacks [38]. The analysis of key space can be from the following angles:

1. Initial value

In [39], it is shown that a difference of the order of $10^{-30}$ in the initial value leads to different values after only 99 iterations. Thus, for this sensitivity order we can have $10^{30}$ possible initial values between 0 and 1. Therefore, increasing the number of decimal places to be supported results in increasing the key space of the desired system and thus increasing its safety.

2. Iterations

In order to improve security, it is necessity to improve the key space; thus the number of iterations of each chaotic system is guaranteed to be 1000 at least [1,24].

3. Parameter extraction

According to the algorithm, the probability of the generation of the integer value sequence is $3.55\times 10^{12}$, the integer is transformed into binary sequence after module 256. When normalizing, the sequence of 15 integer values obtained last time is rearranged, and there are $1.3\times 10^{12}$ possibilities. The process has a total of $1.3\times 10^{12}\times 3.55\times 10^{12}=4.615\times 10^{24}$ possibilities, and the second time has the potential of ${\left(4.615\times 10^{24}\right)}^{2}>2^{128}$. On this basis, the seed parameters $\left(\mu ,x_{0},I\right)$ of the next chaotic system are extracted. It not only expands the key space, but also realizes the independence of pseudo-random sequences.

#### 3.3.2. Resistance to Attack

From the analysis of Section 3.2, it is found that the sequence after rearranging has little correlation with the original sequence generated by the chaotic system, and no statistical characteristics could be found. The attack can be considered from the following aspects:

- Attack the seed parameters
- Attack the sequence generated by the chaotic system
- Attack the sequence after rearrangement

For the first point, in the proposed scheme, the key component s are the initial status value $x_{0}$, the system parameter μ and the number of iterations I, where set $x_{0}\in \left(0,1\right)$, μ∈(3.5699,4) and I>1000. The precision of floating point numbers generated by the chaotic system is $10^{-15}$ [20]. $x_{0}$ can be any one among those $10^{15}$ possible values. Similarly, μ can be any values in the range of $\left(4-3.5699\right)\times 10^{15}$ values. A sufficiently large key space is guaranteed in the proposed PRNG for practical applications under this condition.

For the second point, this attack is based on a known and determined sequence of integer value, due to the complex dynamic behavior of the logistic system, the analysis of the system by Section 3.1 can be concluded that the minor changes in the parameters of the chaotic system will result in a great change [13]. Therefore, the sequence generated by the chaotic system has no statistical properties which means cannot be attacked. This also stems from the excellent performance of the logistic chaotic system.

For the third point, the values generated by the initial chaotic iteration are rearranged, and the correlation between the previous and next chaotic iterations is reduced by extracting the parameters of the previous chaotic system. The output sequence of any secure PRNG must be highly random and completely independent [13]. There are $1.3\times 10^{12}\times 3.55\times 10^{12}=4.615\times 10^{24}$ possibilities as a total during the process of rearrangement and extract, and the second time the attack has the potential of ${\left(4.615\times 10^{24}\right)}^{2}>2^{128}$, which represents an attacker’s nth attack if it is an exhaustive attack is used to share ${\left(4.615\times 10^{24}\right)}^{n}$ possibilities. The key space is the total number of different keys that can be used in the procedure [2]. It is generally believed that the key space $<2^{128}$ is not safe enough [13,40,41]. Consequently, it is difficult to speculate the previous chaotic sequence even attacker obtain the key of the next time.

#### 3.3.3. NIST Analysis

Sequences are evaluated by the statistical test suite NIST. The suite contains a statistical package containing 15 tests to quantify and evaluate the randomness of number sequences generated by cryptographic random or pseudo-random number generators [7,9,13,20]. As shown in Table 8, the evaluation index indicates that the data generated by the PRNG based on the logistic chaotic system is the requirement to meet the randomness.

The detection results show that the frequency is 0.556298, which indicates that the ratio of 0 and 1 in the generated sequence is relatively average [24], compared with 0.635558 in [7], 0.1329 in [16] and 0.629806 in [22], which is better than them respectively.

## 4. Conclusions

This paper proposes a PRNG based on a logistic chaotic system. Aiming at the security of chaotic system parameters in the process of generating pseudo-random number sequences, the floating point numbers generated by initial state chaotic systems are rearranged and extracted. The system parameters of the next chaotic system are generated on this basis. The next chaotic system’s system parameters are extracted from the 15 number sequences generated by the previous time. The rearrangement increases the key space, which makes the system parameters of the chaotic system uncontrollable and has no statistical properties, and key space after increasing makes it difficult for exhaustive attacks. On the one hand, the pseudo-random sequence can be output infinitely, which is strong enough to meet all kinds of encryption needs. On the other hand, due to the perfect performance of the logistic chaotic system, the security of the output sequence is strong. The analysis of the characteristics of the logistic chaotic system and integer value sequence shows that the generated pseudo-random sequence has good randomness and independence from the aspects of the histogram, information entropy, numerical calculation, and proportional value.

## Figures and Tables

**Table 1:** Logistic mapping values.

$x_{i}$	Iterations				
	I=1	I=2	I=50	I=100	I=1000
$x_{0}=0.4000$	0.9120	0.3050	0.4341	0.7967	0.1947
$x_{1}=0.4001$	0.9121	0.3047	0.5385	0.6639	0.2505
$x_{2}=0.4002$	0.9122	0.3045	0.6726	0.9110	0.9343

**Table 2:** Results under different seed parameters.

System Parameters μ	Initial Value $x_{0}$	Number of Iterations I	16 Floating Point Numbers Generated y
3.8001	0.4000	100	0.272365554369460
3.8002	0.4001	110	0.641375560291393
3.8003	0.4002	120	0.939999673380606
3.8003	0.4003	120	0.419646260514604
3.8004	0.4003	120	0.935574949032498
3.8004	0.4004	120	0.246683325821152
3.8004	0.4004	121	0.706230850080309

**Table 3:** Information entropy under different extraction methods.

Extraction Method	Extraction Times			
	$10^{7}$	$2\times 10^{7}$	$5\times 10^{7}$	$10^{8}$
(1, 2, 3, 4)	10.4222	10.4224	10.4221	10.4222
(5, 6, 7, 8)	10.4220	10.4218	10.4223	10.4222
(9, 10, 11, 12)	10.4220	10.4224	10.4218	10.4220
(1, 5, 9, 13)	10.4221	10.4220	10.4219	10.4223
(2, 6, 10, 14)	10.4222	10.4221	10.4221	10.4221
(3, 7, 11, 15)	10.4223	10.4214	10.4222	10.4219

**Table 4:** Experimental samples.

Sample	System Parameters μ	$\mathrm{Initial}\;\mathrm{Value}\;x_{0}$	Number of Iterations I
Sample1	3.8000	0.5000	1000
Sample2	3.9748	0.9734	6376
Sample3	3.6779	0.6942	8459
Sample4	3.7166	0.1674	7348

**Table 5:** Standard deviation of samples.

Sample	Standard Deviation
Sample 1	0.000488336
Sample 2	0. 000308604
Sample 3	0.000435827
Sample 4	0.000373529

**Table 6:** Information entropy of integer value sequence.

Sample	Numbers of Sample Extracted						
	10000	20000	40000	60000	80000	100000	200000
Sample 1	7.9682	7.9681	7.9695	7.9692	7.9688	7.9692	7.9692
Sample 2	7.9939	7.9947	7.9951	7.9952	7.9951	7.9956	7.9955
Sample 3	7.9896	7.9911	7.9911	7.9911	7.9913	7.9911	7.9913
Sample 4	7.9922	7.9929	7.9933	7.9936	7.9936	7.9933	7.9934

**Table 7:** Sensitivity analysis.

${\left(\mu ,x_{0},I\right)}_{1}$	${\left(\mu ,x_{0},I\right)}_{2}$	r/%
(3.8001,0.4000,1000)	(3.8002,0.4001,2000)	51.9231
(3.8002,0.4001,2000)	(3.8003,0.4002,1000)	42.3077
(3.8003,0.4003,1000)	(3.8004,0.4003,1000)	51.9231
(3.8004,0.4003,1000)	(3.8004,0.4004,1000)	43.2692
(3.8004,0.40041000)	(3.8004,0.4004,1000)	44.2308

**Table 8:** NIST (National Institute of Science and Technology) test results.

Test Name	*p*_Value	Result
Approximate Entropy	0.287458	SUCCESS
Block Frequency	0.578344	SUCCESS
Cumulative Sums	0.691934	SUCCESS
FFT	0.402675	SUCCESS
Frequency	0.556298	SUCCESS
Linear Complexity	0.651363	SUCCESS
Longest Run	0.084999	SUCCESS
NonOverlapping Template	0.457732	SUCCESS
Overlapping Template	0.210308	SUCCESS
Random Excursions	0.347548	SUCCESS
Random Excursions Variant	0.219526	SUCCESS
Rank	0.670342	SUCCESS
Runs	0.510265	SUCCESS
Serial(1)	0.512756	SUCCESS
Serial(2)	0.595549	SUCCESS
Universal	0.784275	SUCCESS

## References

[1] Li C., Lin D., Feng B., Lü J., Hao F. (2018). Cryptanalysis of a chaotic image encryption algorithm based on information entropy. IEEE Access.

[2] Xu L., Li Z., Li J., Hua W. (2016). A novel bit-level image encryption algorithm based on chaotic maps. Opt. Lasers Eng..

[3] Flores-Vergara A., Inzunza-González E., García-Guerrero E.E., López-Bonilla O.R., Rodríguez-Orozco E., Hernández-Ontiveros J.M., Cárdenas-Valdez J.R., Tlelo-Cuautle E. (2019). Implementing a Chaotic Cryptosystem by Performing Parallel Computing on Embedded Systems with Multiprocessors. Entropy.

[4] Rezk A.A., Madian A.H., Radwan A.G., Soliman A.M. (2019). Reconfigurable chaotic pseudo random number generator based on FPGA. AEU Int. J. Electron. Commun..

[5] Li S., Ding W., Yin B., Zhang T., Ma Y. (2018). A novel delay linear coupling logistics map model for color image encryption. Entropy.

[6] Natiq H., Said M.R.M., Al-Saidi N.M.G., Kilicman A. (2019). Dynamics and complexity of a new 4d chaotic laser system. Entropy.

[7] Patidar V., Sud K.K., Pareek N.K. (2009). A pseudo random bit generator based on chaotic logistic map and its statistical testing. Informatica.

[8] Behnia S., Akhshani A., Ahadpour S., Mahmodi H., Akhavan A. (2007). A fast chaotic encryption scheme based on piecewise nonlinear chaotic maps. Phys. Lett. A.

[9] Fatemi-Behbahani E., Ansari-Asl K., Farshidi E. (2016). A new approach to analysis and design of chaos-based random number generators using algorithmic converter. Circuits Syst. Signal Process..

[10] López A.B.O., Maranon G.A., Estévez A.G., Dégano G.P., García M.R., Vitini F.M. (2010). Trident, a new pseudo random number generator based on coupled chaotic maps. Computational Intelligence in Security for Information Systems 2010.

[11] Patidar V., Sud K.K. (2009). A novel pseudo random bit generator based on chaotic standard map and its testing. Electron. J. Theor. Phys..

[12] Li X., Zhang G., Liao Y. Chaos-based true random number generator using image. Proceedings of the 2011 International Conference on Computer Science and Service System (CSSS).

[13] François M., Grosges T., Barchiesi D., Erra R. (2014). Pseudo-random number generator based on mixing of three chaotic maps. Commun. Nonlinear Sci. Numer. Simul..

[14] Xu D., Xue X.X., Wang T. (2016). Research on chaotic pseudo random bit generator based on logistic map. Microelectron. Comput..

[15] Zheng F., Tian X.-J., Song J.-Y., Li X.-Y. (2008). Pseudo-random sequence generator based on the generalized Henon map. J. China Univ. Posts Telecommun..

[16] Karakaya B., Gülten A., Frasca M. (2019). A true random bit generator based on a memristive chaotic circuit: Analysis, design and FPGA implementation. Chaos Solitons Fractals.

[17] Oishi S., Inoue H. (1982). Pseudo-random number generators and chaos. IEICE Trans. (1976–1990).

[18] González J.A., Pino R. (1999). A random number generator based on unpredictable chaotic functions. Comput. Phys. Commun..

[19] Wang L., Wang F.P., Wang Z.J. (2006). A novel chaos-based pseudo-random number generator. Acta Phys. Sin..

[20] Wang Y., Liu Z., Ma J., He H. (2016). A pseudo-random number generator based on piecewise logistic map. Nonlinear Dyn..

[21] Pareek N.K., Patidar V., Sud K.K. (2016). A Random Bit Generator Using Chaotic Maps. Nonlinear Dyn..

[22] Liu L., Miao S., Hu H., Deng Y. (2016). Pseudo-random bit generator based on non-stationary logistic maps. IET Inf. Secur..

[23] Elmanfaloty R.A., Abou-Bakr E. (2019). Random property enhancement of a 1D chaotic PRNG with finite precision implementation. Chaos Solitons Fractals.

[24] Kanso A., Smaoui N. (2009). Logistic chaotic maps for binary numbers generations. Chaos Solitons Fractals.

[25] Ulam S.M. (1947). On combination of stochastic and deterministic processes. Bull. Am. Math. Soc..

[26] Murillo-Escobar M.A., Cruz-Hernández C., Cardoza-Avendaño L., Méndez-Ramírez R. (2017). A novel pseudorandom number generator based on pseudorandomly enhanced logistic map. Nonlinear Dyn..

[27] Akhavan A., Samsudin A., Akhshani A. (2009). Hash function based on piecewise nonlinear chaotic map. Chaos Solitons Fractals.

[28] Zhang J., Wang X., Zhang W. (2007). Chaotic keyed hash function based on feedforward–feedback nonlinear digital filter. Phys. Lett. A.

[29] Shuai C., Zhong X.X. (2007). Chaotic block iterating method for pseudo-random sequence generator. J. China Univ. Posts Telecommun..

[30] Saito M., Matsumoto M. (2009). A PRNG specialized in double precision floating point numbers using an affine transition. Monte Carlo and Quasi-Monte Carlo Methods 2008.

[31] Xuan L., Yan J.N. (2009). The “one-group-one-cipher” cryptograph of block-cipher based on chaotic. J. China Inst. Commun..

[32] Tang R., Duan J., Deng H. (2017). Image encryption algorithm based on Logistic chaotic sequence and DES. J. Comput. Appl..

[33] Zhao J., Wang S., Chang Y., Li X. (2015). A novel image encryption scheme based on an improper fractional-order chaotic system. Nonlinear Dyn..

[34] Wang X.Y., Gu S.X., Zhang Y.Q. (2015). Novel image encryption algorithm based on cycle shift and chaotic system. Opt. Lasers Eng..

[35] Pareek N.K., Patidar V., Sud K.K. (2006). Image encryption using chaotic logistic map. Image Vis. Comput..

[36] Pareek N.K., Patidar V., Sud K.K. (2003). Discrete chaotic cryptography using external key. Phys. Lett. A.

[37] Liu H., Wang X. (2010). Color image encryption based on one-time keys and robust chaotic maps. Comput. Math. Appl..

[38] Alvarez G., Li S. (2006). Some basic cryptographic requirements for chaos-based cryptosystems. Int. J. Bifurc. Chaos.

[39] Bose R., Banerjee A. Implementing symmetric key cryptography using chaos functions. Proceedings of the 7th International Conference on Advanced Communications and Computing (ADCOM).

[40] Özkaynak F. (2018). Brief review on application of nonlinear dynamics in image encryption. Nonlinear Dyn..

[41] Özkaynak F., Yavuz S. (2013). Security problems for a pseudorandom sequence generator based on the Chen chaotic system. Comput. Phys. Commun..

